# Important Considerations for Signal Detection and Evaluation

**DOI:** 10.1007/s43441-023-00518-0

**Published:** 2023-04-17

**Authors:** James Buchanan, Mengchun Li

**Affiliations:** 1 Covilance, LLC, 2723 Sequoia Way, Belmont, CA 94002 USA; 2 TB Alliance, New York, NY USA

**Keywords:** Signal detection, Evaluation, Causal association, FDA medical queries, Bradford Hill

## Abstract

Safety clinicians have a wealth of resources describing how to perform signal detection. Nevertheless, there are some nuances concerning approaches taken by regulatory authorities and statistical considerations that should be appreciated. New approaches, such as the FDA Medical Queries, illustrate the value of considering medical concepts over individual adverse events. One area which would benefit from further clarity is how safety signals may be evaluated for evidence of a causal relationship to the drug of interest. Just as such safety signals can take many forms, the types of tools and methods required to interrogate these signals are equally as diverse. An understanding of the complexity of this process can aid the safety reviewer in successfully characterizing the emerging safety profile of a drug during the pre-marketing phase of development.

## Introduction

There are a number of resources available to safety clinicians concerning methods by which safety signals may be identified during clinical development, including a number of publications [1–4], books [5, 6], working groups [7, 8] and guidances from regulatory authorities [9, 10]. In addition to these resources, several important principles are worth bearing in mind to facilitate signal detection which will be topics for discussion in this paper.

Subsequently, having identified that event with an unknown causal relationship to treatment that is recognized as worthy of further exploration and continued surveillance, i.e., the safety signal, an exploration of information that would support (or refute) a causal association to the drug of interest is required. The approach to causal evaluation has included the general application of clinical judgement, and the use of various algorithms and probabilistic approaches, each with a variety of limitations. More specific criteria have been offered by the CIOMS VI Working Group and FDA which can be applied readily within the causality framework described by Bradford Hill. This paper will highlight some important principles to consider during signal detection and summarize a framework to evaluate evidence for a causal association between and adverse event and the drug of interest.

## Considerations for Signal Detection in Clinical Development

### Common Sources of Signals by Organ Systems, Metabolic Enzymes and Transporters

The search for safety signals spans a number of data sources including non-clinical sources (e.g., animal toxicology and safety pharmacology findings) as well as clinical data sources (e.g., adverse events (AEs), laboratory and vital sign data). The search for safety signals can be focused by considering those areas that are the most frequent sources of important product risks, particularly those that lead to removal of marketing authorization. Topics of interest where safety signals of concern may arise are discussed in the FDA’s Pre-Market Risk Assessment guidance [9] and include hepatotoxicity, nephrotoxicity and cardiotoxicity. The Interactive Safety Graphics taskforce of the American Statistical Association Biopharmaceutical Safety Working Group has developed a suite of open-source tools for detecting and evaluating safety signals, including the Hepatic Explorer and will be releasing tools to look for nephrotoxicity and cardiac toxicity this year (see https://safetygraphics.github.io/).

The potential for safety issues to arise from drug interactions with metabolizing enzymes and transporters was highlighted in the FDA guidance for industry “In Vitro Drug Interaction Studies—Cytochrome P450 Enzyme-and Transporter-Mediated Drug Interactions” [11]. In fact, transporter interactions are an under-appreciated source of safety issues. As membrane transporters present in various tissues can have clinically relevant consequences on the pharmacokinetics and pharmacodynamics of a drug, a compound that interacts with these transporters can produce clinically important effects. For example, the uptake of some drugs into the liver is subject to the transporters OATP1B1 or OATP1B3. A drug that inhibits OATP1B1 or OATP1B3 could adversely affect the metabolism of the substrates for these transporters. In addition to transporter inhibition, induction of transporters should also be considered. For example, tienilic acid was marketed as an antihypertensive diuretic drug but was withdrawn due to occurrences of fulminant hepatic failure. In a non-clinical study, it was demonstrated that tienilic acid resulted in enhanced expression of the hepatic transporter multidrug resistance-associated protein 3 (MRP3) which correlated with an increase in serum total bilirubin, suggesting increased bilirubin transport from the liver into the peripheral blood flow [12].

### Aggregate Data Analysis

#### AE Frequency Imbalances

A common approach to the analysis of aggregate AE data is to review a table of AE frequencies by treatment arm. When there is a sizable difference in the frequency of a particular AE between treatment groups, this may rise to the level of a safety signal. However, what magnitude of difference ought to be considered a signal has not been clearly defined and remains a matter of clinical judgment. There are several approaches that can draw the reviewer’s attention to those AE terms with the largest magnitude of difference.

**Figure 1:**
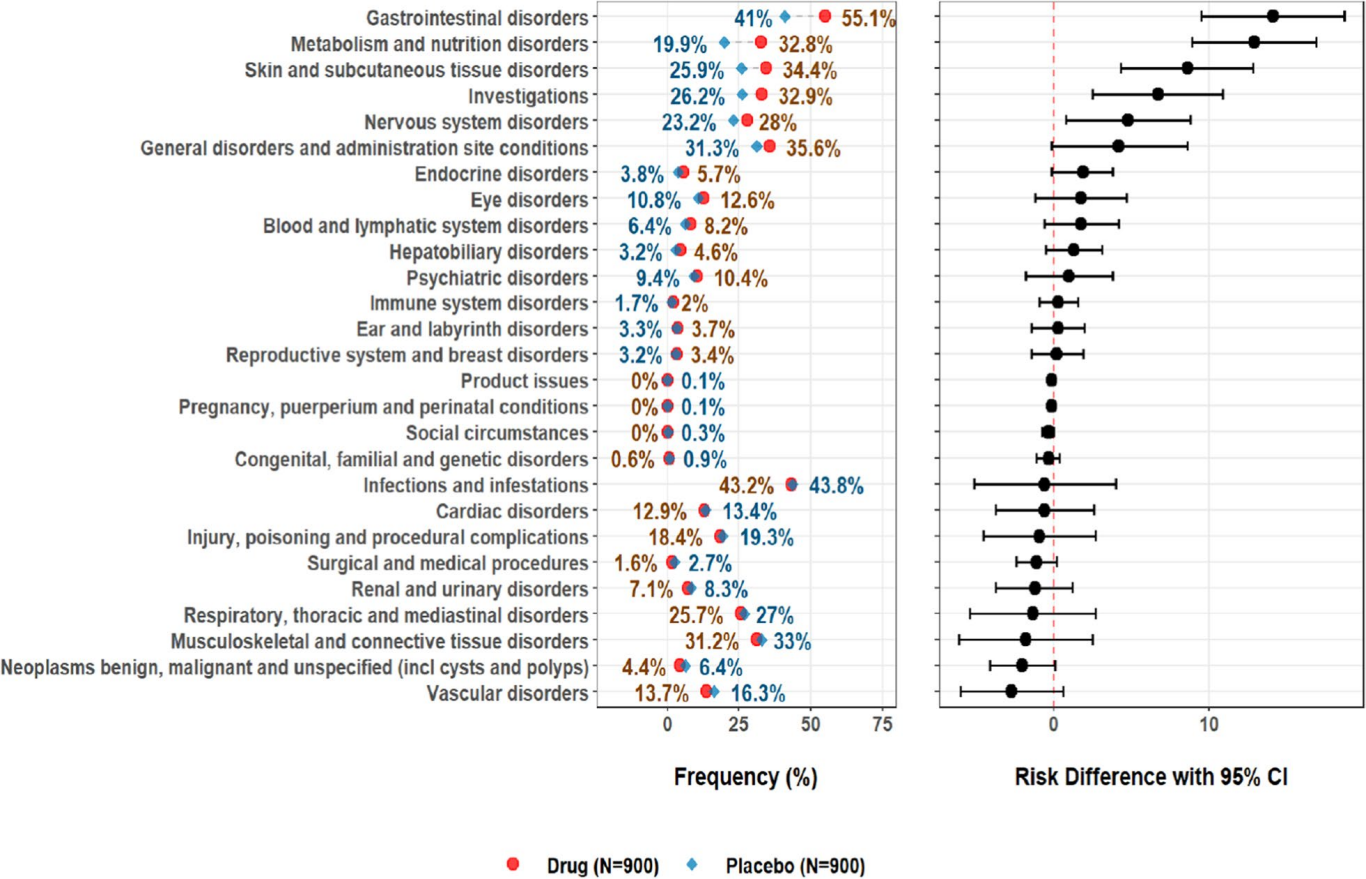
Adverse Events by Frequency and Risk Difference (FDA Advancing Premarket Safety Analytics, Standard Tables and Figures Integrated Guide, September 14, 2022).

Frequency tables can be sorted by the risk difference; that is, the absolute difference between the frequencies of an AE term between treatment groups (Fig. 1). The FDA Medical Reviewer’s Guidance [10] suggests that the reviewer initially focuses on those events with an incidence of ≥ 5% and 2x, or some other percentage, greater than the placebo incidence. Additionally, the risk ratio can also be used as a sorting tool. The risk ratio is the frequency of an AE term in one group (typically the drug treatment arm) divided by the AE frequency in a second group (typically the control arm). For example, if the frequency of tachycardia is 5.3% in the active drug arm and 2.7% in the placebo arm, the risk ratio is 5.3%/2.7% or 2. The risk ratio can be combined with the risk difference to sort the AE frequency table, bringing those events with the greatest magnitude of difference to the attention of the reviewer. For example, in the tafamidis clinical review report [13], the medical reviewer sorted AEs by those with an incidence > 5% with risk difference ≥ 2% and risk ratio ≥ 1.2 compared to the placebo group.

Graphical displays that depict AE frequencies by treatment arm and the corresponding risk differences (Fig. 1) can be sorted on the basis of the magnitude of the risk difference. This allows the reviewer to quickly identify those events showing the greatest differences in frequency between treatment groups. FDA has described their recommendations for standard safety tables and figures in a presentation made in September 2022 [14]. One of the recommended displays is, in fact, a display of AEs and frequency rates in descending order of risk difference. Not only does such a display facilitate review by the safety clinician, focusing attention on those events most likely to represent safety signals, but it also prepares that the company to produce the types of outputs FDA prefers to receive in a marketing application. A number of commercial tools can produce such figures, but open-source tools are also available (https://safetygraphics.github.io/).

**Figure 2:**
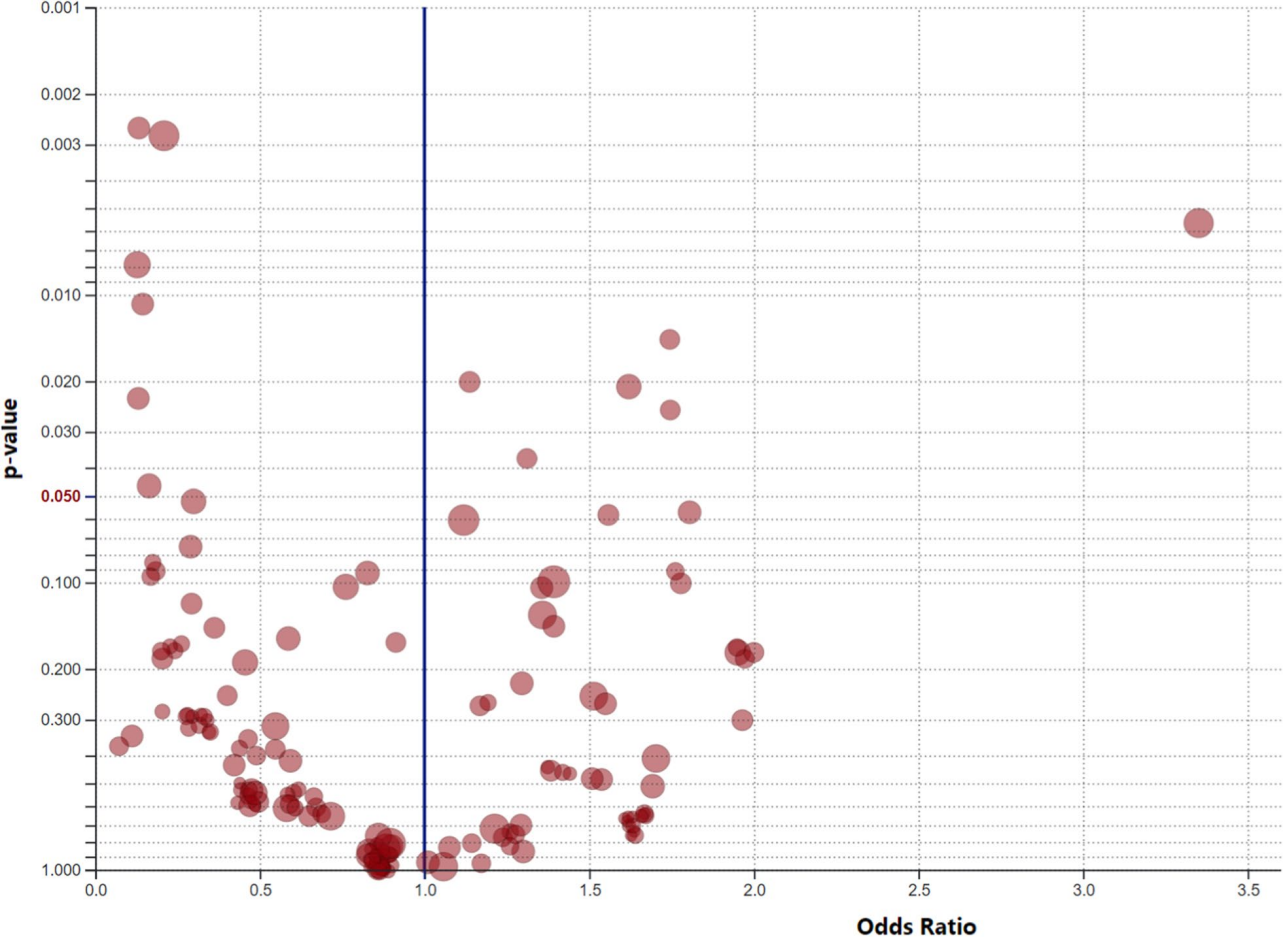
Adverse Event Volcano Plot (https://volcano-plot.s3.amazonaws.com/volcano_plot.html#references).

Volcano plots are another way to easily visualize differences in AE rates between treatment groups (Fig. 2). These displays can also be modified to incorporate statistical measures, such as the odds ratio or hazard ratio. Events with a greater relative frequency in one treatment arm accumulate on one side of the graph while events with a greater relative frequency in another treatment arm accumulate on the other side of the graph. The vertical distance is a measure of the magnitude of difference between treatment arms. The threshold for statistical significance can focus the reviewer’s attention on those events with the greatest magnitude of difference between treatment arms, but caution is always warranted in applying statistical significance measures to post-hoc analyses.

An important consideration in the evaluation of AE frequencies is to not limit the analysis to individual event terms. Although at its inception the MedDRA preferred term (PT) was intended to represent a single medical concept, that has not always been the case. The PTs “abdominal pain”, “abdominal pain upper,” and “abdominal pain lower” are all independent preferred terms, yet all encompass the medical concept of abdominal pain. Thus, a frequency analysis based on a single preferred term may misrepresent the actual frequency of the medical concept it was intended to characterize. One approach to remedy this limitation has been to combine a preferred term from a clinical SOC with a corresponding term from the Investigations SOC, where appropriate. For example, the MedDRA preferred term of “hypokalaemia” from the Metabolism and Nutrition Disorders SOC would be combined with “blood potassium decreased” from the Investigations SOC to better represent the frequency of potassium loss. Of course, the frequencies of these two terms cannot be simply added together since a given subject may have had both terms reported which would lead to double counting.

Combining PTs can be expanded further using an aggregation of multiple relevant preferred terms. Admittedly, Standard MedDRA Queries (SMQs) aggregate multiple PTs into a list of terms representing various medical conditions; however, the large number of included terms render such a tool rather non-specific. Indeed, the MedDRA MSSO states “SMQs may have a mixture of very specific terms and less specific terms that are consistent with a description of the overall clinical syndrome associated with a particular adverse event and drug exposure.^1^” Casting such a broad net may improve sensitivity of finding a condition of interest but at the expense of specificity. A more targeted list of PTs that closely match the medical condition of interest may be more valuable when evaluating relative event frequencies. Such an approach has been recently advocated by FDA using FDA Medical Queries (FMQs), using relatively small aggregates of PTs denoting a common medical concept [14]. For example, the relative frequency of the event “anxiety” does not fully account for other instances of anxiety-related events. The FMQ for anxiety includes the PTs of “anxiety,” “generalized anxiety disorder,” “nervousness,” “panic attack,” and “panic disorder.” An analysis of the relative frequencies of this composite of terms will provide a better estimate of the occurrence of this medical concept rather than a single representation of the overall concept. Differences in event frequencies when using single terms may not be present when an aggregation of terms is used and, similarly, differences not seen using a single term may emerge when the aggregated term approach is used. Like SMQs, the FMQs have narrow and broad versions; however, a finding using the relatively small size of a narrow FMQ is estimated to provide about 90% probability that the medical concept has occurred [14].

An interesting aspect of the FDA’s FMQs is the inclusion of algorithmic FMQs which include other datasets such as laboratory, concomitant medications, and medical history. Additionally, some use temporal associations between these data elements. An example is the Rhabomyolysis FMQ that is defined by the following criteria [14]:

1. Any Rhabdomyolysis FMQ Narrow term

2. Urine myoglobin >ULN^2^

3. CPK^3^ >5 x ULN and NO occurrence of:

- CPK > ULN at baseline OR,
- CPK-MB /CPK > 0.05 with start date within 3 days

4. [PT Myalgia + PT Muscular Weakness + (PT Myoglobin Urine Present OR PT Chromaturia)] with start date within 7 days of each other

The expression of AE frequencies as composites of PTs already appears in the Adverse Reactions section of a number of marketed product labels. With the introduction of FMQs FDA is considering how to incorporate analyses based on FMQs into product labels in the future.

A number of companies already use internally derived combinations of event terms to perform analyses, but the formal release of FDA’s FMQs now provides a more standardized approach to this analysis technique. Hopefully, it will also encourage a move away from safety analysis based solely on “adverse events”, but rather move to thinking about safety in terms of “adverse effects”, where an adverse effect can be characterized by one or more adverse events as well as untoward effects involving laboratory parameters and/or vital signs. Any analysis using a combination of terms, such as FMQs, and across data domains, however, should be approached with appropriate input from biostatisticians.

#### Dose-Response Relationship

An apparent relationship between the extent of drug exposure and the frequency of an AE can indicate a safety signal as well as offer a piece of evidence in support of a causal association. Most commonly single ascending and multiple ascending dose Phase 1 studies are the source of this information, yet Phase 2 studies may investigate multiple doses. In addition to absolute dose, the relationship to pharmacokinetic variables, such as C_max_ and AUC, should be explored. Furthermore, the time of onset of AEs relative to the T_max_ can be another important aspect for causal assessment. The relationship between event frequency and cumulative dose can be evaluated in Phase 2 and 3 studies. Consider too that the extent of drug exposure may also be related to increasing severity of an AE.

#### Treatment Discontinuations

One area of particular concern to FDA has been circumstances that result in a study subject discontinuing the study treatment, notably those that are due to adverse events or perceived poor tolerability of the drug by the study subject. Proper collection of treatment discontinuations due to AEs may be problematic based on the choices provided on the case report form (CRF) and/or instructions to site personnel. Qu et al. [15] described this concern regarding inaccurate collection of the reasons for treatment discontinuation and offered considerations for improvement.

**Figure 3:**
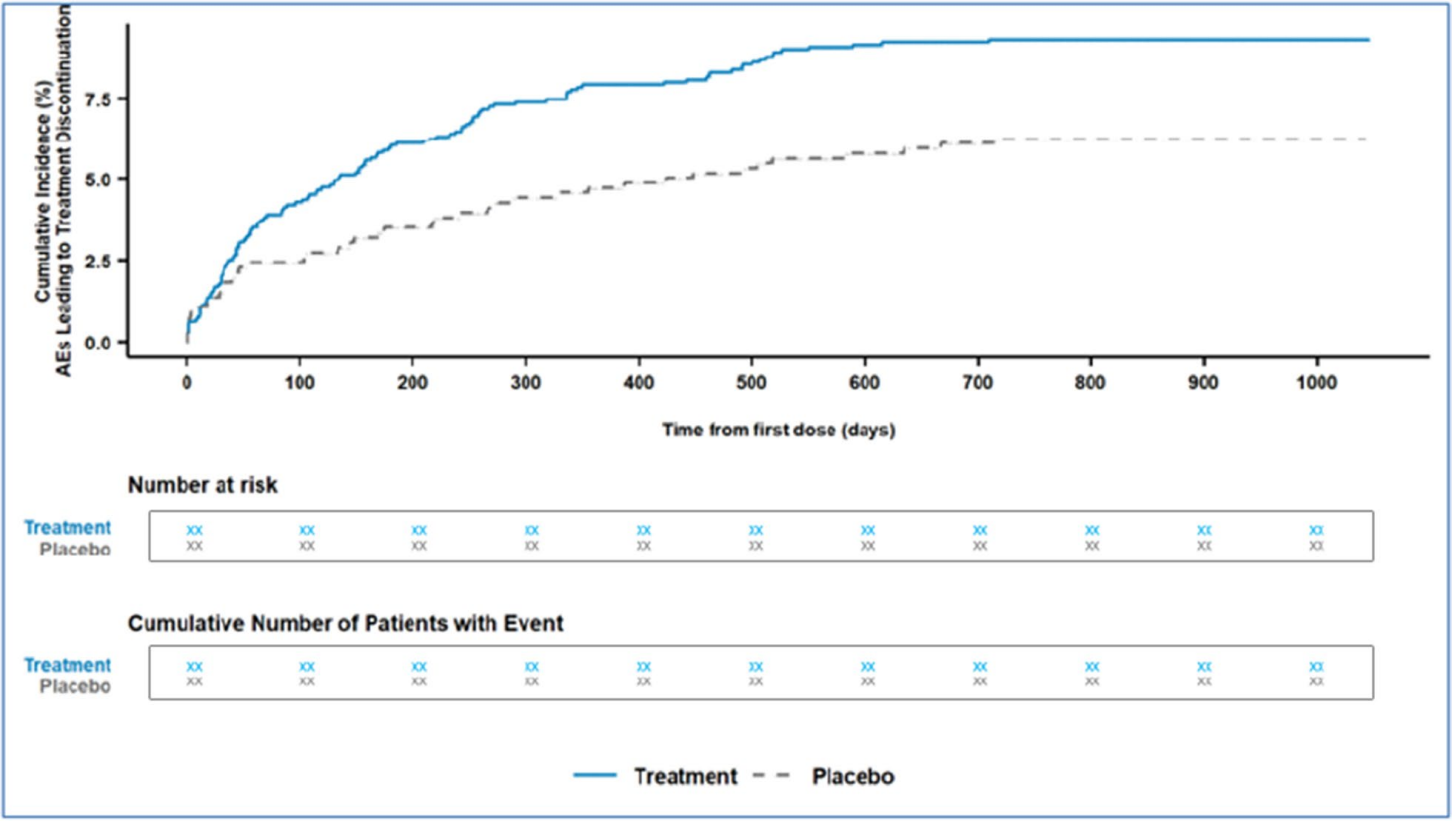
Time to Adverse Event Leading to Treatment Discontinuation.

Assuming that such data have been adequately collected, several analyses may be conducted following recommendations from the FDA Reviewer Guidance [10]: 1) determine if there are clinically meaningful differences in drop-outs between treatment arms and which AEs lead to drop-outs, and 2) is there a dose–response or time-dependency to these drop-out? Also consider that given subject may have discontinued treatment due to 2 or more AEs. Another contributing factor may be the extent that laboratory abnormalities (particularly those not considered adverse events) precipitate study drug discontinuation. A means to display this information graphically is a Kaplan–Meier plot of the cumulative incidence of an AE leading to treatment discontinuation. An example is provided in the FDA’s Integrated Guide to Standard Safety Tables and Figures^4^ and illustrated in Fig. 3.

Early treatment discontinuations preclude the ability to follow the subject for the intended duration of the study. Multiple instances of early treatment discontinuations can create an imbalance in the treatment duration as a whole in that treatment arm compared to other treatment groups. This confounding of the data is further described in “Unequal treatment duration” below.

#### Recurrent Adverse Events

In general, for the purpose of calculating incidences, AE tables count subjects who experience an AE once. Thus, subjects who experience a given AE once are treated the same as subjects who develop multiple recurrences of that AE. This is basically a time-to-first-event analysis. However, all things being equal, multiple occurrences of an AE in a given subject while on drug therapy are more likely drug-related, as described in the FDA’s Reviewer Guidance [10]. Given the regulator’s concern for this issue, it is advisable to identify instances of AEs that occur multiple times in a given subject to determine whether there is an underlying reason for the recurrence; perhaps an unresolved underlying medical condition, or if continued exposure to the study drug produces the recurrent events. Models for evaluating recurrent adverse events exist [16, 17], but the safety clinician is advised to seek the counsel of biostatisticians to develop the most appropriate approach for their data.

### Confounding Considerations

#### Unequal Treatment Duration

The interpretation of adverse event and laboratory data can be confounded when the duration of study drug exposure is not equivalent across treatment groups [18]. The greater the duration of follow-up on-study results in more opportunities to identify adverse events and laboratory abnormalities that can be independent of a drug effect. Alternatively, AEs that lead to study drug discontinuation more commonly in one arm result in a greater duration of on-treatment data collection in the other arm that can result in apparent AE or laboratory abnormality imbalances. When quantifying the frequency of AEs, the standard approach is to calculate the crude rate or incidence proportion (the number of subjects reported to have experienced a specific AE divided by the number of subjects exposed to the drug). However, when the duration of treatment is unequal between treatment groups, this approach can yield misleading results. Before producing the standard AE frequency tables (or composite event frequency tables or laboratory analyte comparisons), careful consideration should be given to determining that the duration of exposure to study treatment is relatively equivalent between groups. If a discrepancy is found, there are statistical methods to account for such differences. One approach is to express event frequencies in a time-adjusted manner, such as events per subject-month or subject-year. This normalizes the treatment duration allowing a more accurate comparison of event rates. However, this approach assumes that the hazard rate for the occurrence of that AE is constant over time, which may not be true.

An example of how differences in treatment duration affect the interpretation of the safety profile is illustrated by a dossier submitted to a regulatory authority for the treatment of medullary thyroid carcinoma with vandetanib [19]. The risk for bias was high as the protocol allowed study subjects to switch treatment upon disease progression. This resulted in the treatment duration for subjects in the vandetanib group to be considerably longer than in the best supportive care group (88.6 weeks vs. 37.1 weeks, respectively). Serious adverse events appeared to occur more frequently in the vandetanib group with an estimated relative risk (RR) of 1.87 with 95% confidence interval (CI) [1.01, 3.48]. This statistically significant result was interpreted as evidence of vandetanib causing greater harm. The analysis was repeated when it was recognized that the greater time on therapy among vandetanib recipients may have biased the results. A non-statistically significant result was found when survival time methods were used, and the hazard ratio (HR) was estimated (HR 1.4, 95% CI: [0.74, 2.63]).

Since inappropriate use of these methods could lead to under- or over-estimating the actual risk of a given AE, it is important for the safety clinician to recognize the circumstances when a standard incidence proportion may not be valid and to seek out expertise from statistical colleagues.

#### Unequal Concomitant Medication Use and Comorbidities

The randomization process for a clinical trial attempts to produce a study population that is relative homogenous across treatment arms. However, there may still be substantial differences in the distribution of baseline medical conditions, the use of medications at baseline, or the use of concomitant medications following randomization that could impact safety evaluation. The unequal distribution of these factors can result in the emergence of AEs resulting from these factors that are also unequally distributed. The resulting apparent AE frequency differences could be erroneously interpreted as a possible study drug effect. Thus, whenever a signal emerges prompted by an AE frequency difference, confounding factors such as these should always be sought. Analyses using stratification are one method to account for this type of confounding. Just as subject characteristics might lead to differences in event frequencies between treatment groups, stratification by one or more of these characteristics may also lead to the emergence of signals that otherwise do not appear in the overall dataset [20]. Exploration in these strata is encouraged as it may lead to identification of populations at particular risk.

#### Unequal Appearance of Post-Randomization Events

Despite randomization, situations may arise after the initiation of study drug that introduce new clinical conditions and/or medication use. For example, if one treatment arm is disproportionately conducted in regions during “flu” season, influenza-related signs and symptoms may be over-represented in that treatment arm. This imbalance is not because the study drug causes influenza, but rather it is due to confounding by the unequal distribution of post-randomization events. Post-randomization events can take several forms. To the safety clinician, these take the form of clinical intercurrent events – the development of a “cold,” the onset of seasonal rhinitis, or a skiing accident that produces a femur fracture. However, in the statistical community, intercurrent events are those that occur after treatment initiation that affect either the interpretation or the existence of the measurements associated with the clinical question of interest. From a safety perspective, these can include early cessation of study drug treatment, an interruption in the collection of data (e.g., missed clinic visits), and the introduction of a new treatment. How such intercurrent events can be managed to aid in the evaluation of safety data is the purpose of the estimand concept. While a discussion of safety estimands is beyond the scope of this discussion, the reader is encouraged to engage their statistical colleagues as well as review several recent references to better understand the application of the estimand framework [21, 22]. In fact, the application of the estimand framework to the analysis of safety data has garnered increasing attention recently. As this is a new, developing area in safety evaluation and a number of working groups are actively exploring how this technique can be applied, the reader can expect more publications on safety estimands in the near future.

### Causality Analysis

A variety of approaches to causality analysis exist, ranging from clinical judgment, through various algorithms to Bayesian methodologies [23]. All these methods have limitations. While clinical judgment suffers from poor inter-rater reliability, algorithms fundamentally use arbitrary scoring systems developed for individual case assessment that is not appropriate for aggregate data evaluation and Bayesian methods have limited practical applicability for most sponsors [24].

#### CIOMS VI and FDA Causality Criteria

There are two useful sources of criteria to assess causal associations when examining aggregate safety data. The CIOMS VI causality criteria [25] span individual case assessment, aggregate data assessment and previous knowledge of the adverse event or drug class. In terms of aggregate data assessment, CIOMS recommends considering the following 7 criteria: 1) is the event a recognized consequence of overdose with the drug, 2) is the event rare among untreated patients, 3) is the event commonly drug-related, 4) is there pharmacokinetic evidence to support an association (including drug interactions), 5) is there a known mechanism of action that could produce the event, 6) is the event a recognized effect of other drugs in the class, and 7) were similar findings found in animal and non-clinical studies. Secondly, the FDA’s draft guidance for Sponsor Responsibilities-Safety Reporting Requirements and Safety Assessment for IND and Bioavailability/Bioequivalence Studies [26] lists 9 factors to consider when assessing whether there is a reasonable possibility that a drug is causally associated with an adverse event: 1) extent of the increase in incidence (of the event) seen in the test group compared to the control groups, 2) evidence of a dose-response relationship, 3) temporal relationship, 4) consistency of the increased incidence of the event in the test group in multiple trials, 5) presence of a plausible mechanism of action, 6) non-clinical evidence to support the finding, 7) pharmacology of the drug and known class effects that support a relationship, 8) pattern of the event across the study population (e.g., higher frequency in susceptible groups), and 9) occurrence of other potentially related adverse events (e.g., occurrence of transient ischemic attacks with strokes, occurrence of elevated creatine kinase with rhabdomyolysis).

#### Bradford Hill Causality Criteria Framework

In 1965, Sir Austin Bradford Hill introduced his framework of evidence to consider when determining if an observed association can be concluded to be causative [27]. The example he presented was the risk of smoking causing cancer, yet since then the methodology has shown itself to also work well for causal assessments of drugs with adverse events.

There are 9 classes of criteria to consider in a causal evaluation (Table 1):

**Table 1:** Bradford Hill Criteria of Causation

Criteria class	Description
Strength of Association	This refers to statistical evidence of an association between a drug and an AE. For example, the difference in AE frequencies between a drug treatment group and control arm may be described by an odds ratio or a hazard rate
Consistency	Are there similar findings across different groups? This could refer to different clinical trials, different patient populations, or different global regions, for example. Commonly in clinical development, consistency would be assessed as the extent to which the safety finding was evident in multiple clinical trials of a drug
Specificity	This criterion is difficult to apply in drug safety evaluations. The intent is that if a drug produces a specific effect not otherwise encountered with other drugs, that lends credence to a causative role. Few examples exist in drug safety, but the relationship between quinolone antibiotics and tendon rupture, and practolol with sclerosing peritonitis are two examples
Temporality	A short time to onset between drug exposure and the appearance of the AE is more suggestive of a causative role than a long delay would suggest. Positive dechallenge and positive rechallenge can also be considered in this criterion
Biological gradient	This refers to a dose-response relationship. Apart from dose per se, consider too a relationship with cumulative dose as well as pharmacokinetic factors such as Cmax and AUC
Plausibility	Is there a credible mechanism of action that could explain how the drug could produce the AE?
Coherence	Like specificity, this criterion is difficult to apply in drug safety evaluations and is also the most misunderstood based on published evaluations. Consider “coherence” to be the inverse of “plausibility.” While “plausibility” starts with the drug’s mechanism of action and asks if that is consistent with the production of the AE, “coherence” instead starts with the known etiologies of the AE and asks if the drug’s effect is consistent with these. For example, hyperinsulinemia can result in hypoglycemia and associated symptoms. Drugs that result in increased insulin secretion could produce hypoglycemia
Experiment	This refers to non-clinical data that may support the clinical data. This could be animal toxicology studies or in vitro tests. Not uncommonly in the literature this criterion has been mistaken to mean other clinical studies
Analogy	Are there analogous examples of similar drugs causing a similar AE, such as known class effects?

Specific causality criteria proposed by CIOMS and FDA fit quite well within the Bradford Hill framework as indicated in Table 2.

**Table 2:** CIOMS and FDA Causality Criteria within the Bradford Hill Framework

Bradford Hill Framework	CIOMS Criteria	FDA Criteria
Strength of association	• Positive outcome in targeted safety study(ies) • Consistently higher incidence vs comparator, including discontinuations due to the AE, earlier onset, greater severity	• Extent of the increase in incidence seen in the test group compared to the control group • Occurrence of other potentially related adverse events
Consistency	• Consistent pattern of presenting symptoms • Consistent time to onset • Consistent trends across studies	• Consistency of the increase in multiple trials • Pattern across the study population (e.g., at risk populations)
Temporality	• Time to onset plausible • Positive dechallenge/rechallenge	• Temporal relationship
Biological gradient	• Positive dose-response relationship • Recognized consequence of overdose	• Evidence of a dose-response
Plausibility	• Known mechanism • Pharmacokinetic evidence (including interactions)	• Presence of a plausible mechanism of action • Pharmacology of the drug
Experiment	• Similar findings in animal or in vitro models	• Nonclinical evidence

Based on the how the available data contribute to each of these criteria, some of which may support a causal association while others may not, the reviewer would make a considered judgment as to which direction the balance of the information weighs. Interestingly, when the FDA articulated the factors to consider when deciding if there is a reasonable possibility that a drug caused an AE [26], the choice of factors each fit into this Bradford-Hill framework.

The interested reader is referred to several publications utilizing the Bradford-Hill criteria to assess the causative role of drugs with various risks. In some cases, the evidence was found to support the causative role for the drug [28], while in other cases, the evidence was found lacking [29].

## Conclusion

The characterization of the emerging safety profile of an investigational drug requires an appreciation for the diverse sources and ways in which a signal may arise. The routine use of various analytical tools, including data visualization approaches, can aid the reviewer in both the identification and evaluation of safety signals. Regulatory authorities have offered considerations for data displays and analyses that the safety reviewer can incorporate in the process of routine safety monitoring. A number of statistical considerations should be born in mind during safety data review that may affect the conclusions drawn from these analyses, including differences in treatment duration and imbalances in underlying subject characteristics. Partnership with biostatisticians is an important component of the safety review process to maximize the utility of the accumulating safety data. Having identified a safety signal worthy of further evaluation, a process is required to weigh the evidence supporting or refuting a causal association. The conclusion of a causal association between the drug and an event can be most consistently achieved when clinical judgment is applied to the various criteria offered by CIOMS and FDA within a framework such as offered by Bradford Hill.

## Footnotes

1 Introductory Guide for Standardised MedDRA Queries (SMQs) Version 25.0, March 2022.

2 ULN = upper limit of normal.

3 CPK = creatine phosphokinase.

4 https://www.regulations.gov/document/FDA-2022-N-1961-0002

## References

[1] van Manen RP, Fram D, DuMouchel W (2007). Signal detection methodologies to support effective safety management. Expert Opin Drug Saf.

[2] Perez C, Olivier P, Lortal B (2018). Detection of drug safety signals from clinical trials data: role of SUSARs. Pharmacol Res.

[3] Zink RC, Marchenko O, Sanchez-Kam M (2018). Sources of safety data and statistical strategies for design and analysis: clinical trials. Ther Innov Reg Sci.

[4] Buchanan J, Li M, Ni X, Wildfire J (2021). A new paradigm for safety data signal detection and evaluation using open-source software created by an interdisciplinary working group. Ther Innov Reg Sci.

[5] Doan T, Lievano F, Bhattacharya M, Scarazzini L, Renz C, Doan T, Lievano F, Bhattacharya M, Scarazzini L, Renz C (2019). Pharmacovigilance: a practical approach.

[6] Buchanan J, Li M, Wang W, Munsaka M, Buchanan J, Li JX (2022). Safety signaling and causal evaluation. Quantitative Drug Safety and Benefit-Risk Evaluation.

[7] Practical Aspects of Signal Detection in Pharmacovigilance, Report of the CIOMS VIII Group, Geneva, 2010.

[8] Management of Safety Information from Clinical Trials, Report of the CIOMS Working Group VI, Geneva 2005.

[9] Guidance for Industry: Premarket Risk Assessment. FDA. March 2005. https://www.fda.gov/media/71650/download.

[10] Reviewer Guidance: Conducting a Clinical Safety Review of a New Product Application and Preparing a Report on the Review. FDA. February 2005. https://purl.fdlp.gov/GPO/LPS116705.

[11] Guidance for Industry: In Vitro Drug Interaction Studies — Cytochrome P450 Enzyme- and Transporter-Mediated Drug Interactions. FDA. January 2020. https://www.fda.gov/media/134582/download.

[12] Nishiya T, Kataoka H, Mori K (2006). Tienilic acid enhances hyperbilirubinemia in Eisai hyperbilirubinuria rats through hepatic multidrug resistance–associated protein 3 and heme oxygenase-1 induction. Toxicol Sci.

[13] FDA Clinical Review. Tafamidis. April 5, 2019. https://www.accessdata.fda.gov/drugsatfda_docs/nda/2019/211996Orig1s000,%20212161Orig1s000MedR.pdf

[14] FDA Advancing Premarket Safety Analytics. September 14, 2022. https://healthpolicy.duke.edu/events/advancing-premarket-safety-analytics

[15] Qu Y, White RD, Ruberg SJ (2022). Accurate collection of reasons for treatment discontinuation to better define estimands in clinical trials. Ther Innov Regul Sci.

[16] Amorim LDAF, Cai J (2015). Modelling recurrent events: a tutorial for analysis in epidemiology. Int J Epidemiol.

[17] Hengelbrock J, Gillhaus J, Kloss S, Leverkus F (2016). Safety data from randomized controlled trials: applying models for recurrent events. Pharmaceut Statist.

[18] Stegherr R, Beyersmann J, Jeh V (2021). Survival analysis for AdVerse events with VarYing follow-uptimes (SAVVY): rationale and statistical concept of a meta-analytic study. Biometrical J.

[19] Bender R, Beckmann L, Lange S (2016). Biometrical issues in the analysis of adverse events within the benefit assessment of drugs. Pharmaceut Statist.

[20] Osokogu OU, Dodd C, Pacurariu A (2016). Drug safety monitoring in children: performance of signal detection algorithms and impact of age stratification. Drug Saf.

[21] Unkel S, Amiri M, Benda N (2019). On estimands and the analysis of adverse events in the presence of varying follow-up times within the benefit assessment of therapies. Pharmaceut Statist.

[22] Phillips A, Clark T (2021). Estimands in practice: bridging the gap between study objectives and statistical analysis. Pharmaceut Statist.

[23] Agbabiaka TB, Savovic J, Ernst E (2008). Methods for causality assessment of adverse drug reactions: a systematic review. Drug Saf.

[24] Hire RC, Kinage PJ, Gaikwad NN (2013). Causality assessment in pharmacovigilance: a step towards quality care. J App Med Sci.

[25] Management of Safety Information from Clinical Trials: Report of CIOMS Working Group VI. Report of CIOMS Working Group VI. Geneva, 2005. (see Appendix 7) https://cioms.ch/publications/product/management-of-safety-information-from-clinical-trials-report-of-cioms-working-group-vi/#.

[26] FDA Guidance for Industry: Sponsor Responsibilities— Safety Reporting Requirements and Safety Assessment for IND and Bioavailability/Bioequivalence Studies. 2021. https://www.fda.gov/media/150356/download

[27] Hill BA (1965). The environment and disease: association or causation?. Proc Roy Soc Med.

[28] Perrio M, Voss S, Shakir SAW (2007). Application of the Bradford Hill criteria to assess the causality of cisapride-induced arrhythmia. Drug Saf.

[29] Villafuerte-Galvez JA, Kelly CP (2018). Proton pump inhibitors and risk of Clostridium difficile infection: association or causation?. Curr Opin Gastroenterol.

